# Profiling unauthorized natural resource users for better targeting of conservation interventions

**DOI:** 10.1111/cobi.12575

**Published:** 2015-08-03

**Authors:** Mariel Harrison, Julia Baker, Medard Twinamatsiko, E.J. Milner‐Gulland

**Affiliations:** ^1^ Department of Life SciencesImperial College LondonSilwood Park Campus, Buckhurst RoadAscotBerkshireSL5 7PY; ^2^ Parsons Brinckerhoff6 Devonshire SquareLondonEC2M 4YE; ^3^ International Institute for Environment and Development80–86 Gray's Inn RoadLondonWC1×8NH; ^4^ Institute of Tropical Forest ConservationP.O. Box 44KabaleUganda

**Keywords:** mountain gorillas, natural resource use, poaching, poverty, resentment, unmatched count technique, Uganda, caza furtiva, gorilas de montaña, pobreza, resentimiento, técnica de conteo no pareado, Uganda, uso de recursos protegidas

## Abstract

Unauthorized use of natural resources is a key threat to many protected areas. Approaches to reducing this threat include law enforcement and integrated conservation and development (ICD) projects, but for such ICDs to be targeted effectively, it is important to understand who is illegally using which natural resources and why. The nature of unauthorized behavior makes it difficult to ascertain this information through direct questioning. Bwindi Impenetrable National Park, Uganda, has many ICD projects, including authorizing some local people to use certain nontimber forest resources from the park. However, despite over 25 years of ICD, unauthorized resource use continues. We used household surveys, indirect questioning (unmatched count technique), and focus group discussions to generate profiles of authorized and unauthorized resource users and to explore motivations for unauthorized activity. Overall, unauthorized resource use was most common among people from poor households who lived closest to the park boundary and farthest from roads and trading centers. Other motivations for unauthorized resource use included crop raiding by wild animals, inequity of revenue sharing, and lack of employment, factors that created resentment among the poorest communities. In some communities, benefits obtained from ICD were reported to be the greatest deterrents against unauthorized activity, although law enforcement ranked highest overall. Despite the sensitive nature of exploring unauthorized resource use, management‐relevant insights into the profiles and motivations of unauthorized resource users can be gained from a combination of survey techniques, as adopted here. To reduce unauthorized activity at Bwindi, we suggest ICD benefit the poorest people living in remote areas and near the park boundary by providing affordable alternative sources of forest products and addressing crop raiding. To prevent resentment from driving further unauthorized activity, ICDs should be managed transparently and equitably.

## Abstract (es)

Identificación del Perfil de Usuarios No Autorizados de Recursos Naturales para Mejorar las Intervenciones de Conservación

**Resumen**

El uso no autorizado de recursos naturales es una amenaza clave para muchas áreas naturales. Los métodos para la reducción de esta amenaza incluyen la aplicación de leyes y los proyectos integrados de conservación y desarrollo (ICD), pero para que los ICD sean operados efectivamente, es importante comprender quien esta utilizando ilegalmente cuales recursos naturales y porque. La naturaleza de la conducta no autorizada dificulta la determinación de esta información mediante preguntas directas. El Parque Nacional Impenetrable Bwindi, Uganda, tiene muchos proyectos ICD, incluyendo la autorización para que algunos habitantes locales utilicen ciertos recursos no maderables del parque. Sin embargo, no obstante más de 25 años de ICD, el uso no autorizado de recursos continua. Utilizamos muestreos en hogares, entrevistas indirectas (técnica de conteo no pareado) y discusiones con grupos focales para generar perfiles de los usuarios autorizados y no autorizados y para explorar las motivaciones para las actividades no autorizadas.

En general, el uso no autorizado de recursos fue más común entre gente de hogares empobrecidos que vivían cerca del límite del parque y más lejos de los caminos y de los centros de comercio. Otras motivaciones para el uso no autorizado de recursos incluyen la afectación de cultivos por animales silvestres, la desigualdad de la repartición de ganancias y la falta de empleo, factores que crearon resentimiento en las comunidades más pobres. En algunas comunidades, los beneficios obtenidos de los ICD fueron reportados como los mayores factores disuasorios de actividades no autorizadas, aunque la aplicación de leyes fue el más alto en general. A pesar de la naturaleza sensible de la exploración del uso no autorizado de recursos, se puede obtener una perspectiva relevante para el manejo de usuarios no autorizados de recursos por medio de una combinación de técnicas de muestreo, como en este trabajo. Para reducir la actividad no autorizada en Bwindi, sugerimos que los ICD beneficien a la gente más pobre que vive en áreas remotas y cerca de los límites del parque proporcionando fuentes alternativas de productos forestales y atendiendo la afectación de cultivos. Para prevenir que el resentimiento motive mayores actividades no autorizadas, los ICD deben ser manejados transparente y equitativamente.

## Introduction

Protected areas have long been recognized as a vital conservation tool, yet their effectiveness is often threatened by unauthorized resource use (Pfeifer et al. 2012). In the developing world, where the immediate natural environment is frequently the primary provider of food, shelter, and income, local people often have little choice but to enter protected areas to meet basic needs (Blomley et al. 2010). Unauthorized activity has traditionally been addressed through the development and enforcement of regulations governing access to conservation areas. This potentially disadvantages the poorest people living around the protected area and is therefore often ineffective and unethical (Chan et al. 2007).

Integrated conservation and development (ICD) arose in response to the failure of so‐called fortress conservation to reduce anthropogenic pressure on the environment (Hughes & Flintan 2001). A typology of the complex relationship between poverty and conservation includes the following perspectives: poverty and conservation are separate realms; poverty constrains conservation and therefore poverty reduction is a conservation tool; conservation should not compromise poverty reduction out of moral obligation; and poverty reduction depends on living resource conservation, so conservation is a tool for poverty reduction (Adams et al. 2004). The Fifth World Parks Congress in 2003 declared that “protected areas should strive to contribute to poverty reduction at the local level, and at the very minimum must not contribute to or exacerbate poverty.”

Despite the hopes for ICD, the goals of conservation and development often conflict (Campbell et al. 2010), and there is limited evidence of success in achieving both (Davies et al. 2013). This is partly due to poor monitoring and evaluation of projects, meaning success is difficult to measure (Davies et al. 2013). Additionally, ICD projects may be failing because the incentives they offer are inappropriate, too low (Winkler 2011), or do not reach the right people (Blomley et al. 2010).

When designing ICD interventions aiming to reduce unauthorized activity, the first step should be to understand the profiles and motivations of resource users (i.e., who is involved and why). However, unauthorized activity is rarely investigated due to the difficulty of obtaining reliable information on sensitive behavior. When profiles of unauthorized resource users have been generated, researchers have relied on anecdotal evidence (e.g., Kühl et al. 2009), participant observation (e.g., Robbins et al. 2009), or on admissions by interviewees (e.g., Williams et al. 2012) who may respond dishonestly to protect themselves or others. Indirect questioning can be used to complement the above techniques. They generate higher estimates of unauthorized activity than direct questioning (Nuno et al. 2013) and can be used to generate socioeconomic profiles of those likely to be involved (Razafimanahaka et al. 2012; St John et al. 2012). The unmatched count technique (UCT) (Droitcour et al. 1991) has been applied successfully to the illegal killing of birds in Portugal (Fairbrass 2012) and bushmeat hunting in the Serengeti, with participants reporting feeling comfortable answering the questions (Nuno et al. 2013). We used information on the prevalence and socio‐demographic characteristics of unauthorized resource users, data sets on known authorized and unauthorized resource users, and qualitative understanding of motivations behind unauthorized resource use to produce detailed profiles of people who use resources without authorization and to provide information on why this occurs.

In Bwindi Impenetrable National Park (hereafter Bwindi), southwestern Uganda, ICD projects have been implemented for over 25 years, initially in an attempt to reduce local conflict (Laudati 2010; Baker et al. 2013). Bwindi's Multiple Use Program (MUP), formally initiated in 1994, allows locally elected community members (authorized resource users [ARUs]) to harvest monitored quantities of certain resources from inside Bwindi in designated multiple use zones (MUZs) (Wild & Mutebi 1996). Despite this ICD program and many others, unauthorized resource use continues (Blomley et al. 2010). To inform targeting of such conservation interventions, we used law enforcement data, MUP user records, household surveys, and focus groups to determine the resources most commonly extracted from Bwindi; the socioeconomic profiles of authorized and unauthorized resource users; and the motivations behind and deterrents to unauthorized resource use.

## Methods

### Study Site

Bwindi was first designated as a forest reserve in 1932 and as a game reserve in 1961 before being gazetted a national park in 1991 (Plumptre et al. 2004). Bwindi protects a 330.8 km^2^ fragment of afromontane forest that is highly diverse and rich in endemic flora and fauna (Tukahirwa & Pomeroy 1993). Bwindi is home to around half the world's population of approximately 880 critically endangered mountain gorillas (*Gorilla beringei* ssp. *beringei*) ( IGCP 2014 *a*), which in Bwindi are threatened by habitat loss, disease, and accidental trapping in snares set for bushmeat (McNeilage et al. 2007; IGCP 2014 *b*). Bwindi is surrounded by one of the most densely populated areas of rural Africa, with up to 300 people km^−2^, >95% of whom rely on subsistence farming (Plumptre et al. 2004). The loss of income and livelihoods resulting from the designation of the national park led to violent conflict between local people and the Uganda Wildlife Authority (UWA) (Baker et al. 2011). In an attempt to re‐establish a local sense of forest ownership and reduce conflict, a Multiple Use Program (MUP) was introduced that allowed ARUs to harvest honey, basketry materials, wild yams, and medicinal plants inside Bwindi (Wild & Mutebi 1996). The MUP is not the only ICD project at Bwindi. Other projects have included on‐farm tree planting and agricultural support, employment in tourism enterprises, and development projects funded through tourism revenue sharing (Blomley et al. 2010). In Ugandan national parks, 20% of park entrance fees are shared with neighboring communities to support development projects. Because the majority of Bwindi's tourism revenue is generated through the sale of gorilla viewing permits, a portion of each permit fee (US$5 at the time of research) is also shared (Tumusiime & Vedeld 2012). Despite the investment in ICD at Bwindi over the past 20 years, unauthorized resource use continues, albeit at a lower level than before gazettement (Blomley et al. 2010). Resource extraction appears to have shifted from commercial to subsistence use: instances of timber removal and gold mining are rare, but bushmeat hunting continues (McNeilage et al. 2007).

### Data Collection

We adhered to the ethical principles of the International Institute for Environment and Development and had approval from the Uganda Wildlife Authority to conduct our study (see details in Supporting Information). We classified people into 3 categories: unauthorized resource users (URUs) identified from UWA's records of people apprehended for illegal activity in the park; ARUs currently permitted to harvest some resources in the Park under the MUP; and the general population, based on a stratified random sample of people living in the same villages who had not been apprehended for unauthorized activity within the 9 months prior to survey. We conducted household surveys with each of these groups to obtain socio‐demographic information. We estimated the prevalence of use of key natural resources with UCT. We used focus group discussions with local community groups to identify the motivations for unauthorized use of different resources and to triangulate our findings from the broad survey (Gavin et al. 2010).

To select ARUs for interview, UWA's list of MUP members was verified with representatives of local resource user groups, of which every ARU is a member. From 461 verified ARUs, 72 were randomly selected for interview. To identify URUs, we examined law enforcement records from January 2011 to August 2012 with the chief warden for law enforcement and identified individuals apprehended for bushmeat hunting. With assistance from the community conservation warden and rangers, we determined the current residence of these individuals and interviewed all but 1 of the 42. Law enforcement rangers and community conservation rangers collected data on people arrested for any unauthorized activity in Bwindi from August 2012 to July 2013, including name, village, and location of and reason for arrest. Of the 40 people arrested, 25 were Ugandan and resident in a parish bordering Bwindi. We interviewed 12 of these 25.

To sample the general population, we used lists provided by village leaders of households in villages where interviewed resource users lived and randomly selected 2 households for every 1 resource user household in a given village (*n* = 192). In 3 areas of high density of the Batwa indigenous group, we selected 48 Batwa households at random from lists provided by village leaders and conducted interviews of these households to ensure that the views of this often‐marginalized ethnic group were included. This took the general population sample to 240. In total, we surveyed 365 households in 54 villages across 19 of the 23 parishes bordering Bwindi. After being briefed on the purpose of the research, interviewees were asked for their consent to be interviewed. They were not, however, told why they specifically had been selected for interview in order not to introduce strategic bias into responses by members of the URU and ARU samples and to avoid the risk of social stigma if other community members wondered why they had been selected.

The household survey contained questions on ethnicity, number of household members, proximity to roads and trading centers, education of household head, knowledge and perception of ICD projects, and wealth. A household wealth score was calculated from observations of homestead size, structure, and facilities, following focus group discussions with local people to determine what features were associated with different levels of wealth. We took the GPS location of each household interviewed and calculated the straight‐line distance to the Park boundary in ArcGIS (ArcMapTM version 10.0).

Based on key informant interviews, the main Park resources used by local people were bushmeat, firewood, building poles, medicinal plants, and honey. Only the last two of these can be harvested legally from Bwindi and only then by ARUs under MUP rules. We used UCT to estimate the prevalence of each resource use within particular demographic groups. The UCT could not give an estimate of resource use across the wider population, however, because our sampling, though random within a village, was structured around the locations of ARUs and known URUs and purposively included randomly selected Batwa respondents.

Focus group discussions with 17 community groups known as stretcher groups (Supporting Information) were used to verify findings on use of forest resources and to determine motivations for and deterrents against resource use. The discussions dealt with the questions of what motivates people to collect resources from Bwindi and what stops people from doing so. Responses to each question were written and then ranked by participants according to the number of people within the community who they thought were motivated or deterred by the factor. Responses were read for illiterate members. Focus group discussions specifically about bushmeat hunting were also held with the three Reformed Poacher's Associations established around Bwindi at the time of research and followed a similar format.

### Data Analyses

We estimated the prevalence of resource use with separate general linear models (GLMs) for each resource and fitted UCT response to card type (control or treatment [Supporting Information]). For the more commonly used resources, for which statistical models could be generated (bushmeat and firewood), we constructed full models based on a priori hypotheses about variables that could potentially influence resource use (Supporting Information). We undertook a stepwise model simplification based on variable significance but retained variables if their interactions with card type were potentially significant. After model simplification, we obtained a set of models with ΔAICs<4 and then used model averaging in the dredge function in the MuMln package of R (version 2.15.1) to obtain estimates of variable importance and averaged coefficients.

To create profiles of known resource users, we compared the socio‐economic profiles of ARUs (n = 72) and URUs (n = 36) with those of the general population sample (n = 240). The three groups were non‐overlapping. Continuous and categorical variables were analyzed using GLMs and the χ^2^ test respectively. We also created a profile of bushmeat hunters apprehended between January 2011 and July 2013 (n = 46).

Qualitative data from open‐ended questions in the household survey and from focus groups were indexed using hierarchical coding. Analyses using these codes were conducted in Excel.

For each focus group, we calculated a salience score (Papworth et al. 2013) for each motivation for and deterrent to unauthorized resource use:

(1) salience =1+ lengt hi− positio ni lengt hi,

where length is the number of motivations or deterrents given by focus group *i* and position is the rank given to that motivation or deterrent. The cultural salience score (*S*) for each motivation and deterrent was calculated as:

(2) S=∑ salienc ein,

where *n* is the number of focus groups. Salience scores range from 0 to 1; 1 means the item ranked first in every focus group. A low salience score indicated the item was ranked low or was not included in every list and therefore indicates lower importance. Salience scores were used to illustrate the relative importance of the many motivations and deterrents. They were not intended to quantify motivations, but to group them for further analysis.

## Results

### Geographical Variation in Demographic Characteristics

All respondents lived within 6 km of the park boundary (mean = 1.31, n = 362). Mean education of respondents was 3.8 years (n = 357). People living within 0.5 km of Bwindi or over an hour from a road or trading center had significantly fewer years of education and significantly lower wealth scores than average (Supporting Information). Focus groups indicated that crop raiding influenced these relationships; people farming closest to the boundary lose crops and livestock and therefore income to Park animals. Farming was the primary income source for 67% of households surveyed and in the top 3 sources for 98% of households. Many households relied directly on farming for food. Focus groups also reported that when households guard their crops against wild animals, they cannot invest time in other income‐generating activities or send their children to school as regularly.

### Forest Resources Used

Bushmeat was the most commonly used park resource; 26% (SE 8) of interviewed households had consumed it in the previous year and “to hunt bushmeat for food” had the highest salience of all motivations for resource use (Table 1). The next most commonly used Park resource was firewood, followed by medicinal plants, honey, and building poles. The ranking of resource use motivations by focus groups matched the UCT findings, confirming Bwindi resources extracted and their relative importance. Focus groups suggested basketry materials were harvested at a level similar to honey and medicinal plants, but these resources were not included in the UCT analysis because key informants previously indicated that few people used them.

**Table 1:** Prevalence of and reasons for resource use in the surveyed population around Bwindi based on the unmatched count technique (UCT) and focus group discussions

	Legality of	Percent			
	harvest in	prevalence^a^			
Resource	national park	(SE, *p*)	UCT activity	Salience^b^, ^c^	Explanation^c^
Bushmeat	illegal for all	26.0 (8.1, 0.001)	bushmeat consumption	0.629 (a) 0.296 (b) 0.098 (c)	sought as (a) food for household consumption, (b) to sell (c) for medicinal purposes
Firewood	illegal for all	19.6 (8.5, 0.021)	using firewood from the park for cooking	0.478	collected for household use
Medicinal plants	illegal for majority; 130 people authorized to harvest (3.9% of total sample)	15.6 (9.3, 0.094)	using herbs from the park as medical treatment	0.482 (a) 0.402 (b) 0.051 (c) 0.041 (d) 0.017 (e)	collected because they (a) work better than modern healthcare, (b) only grow in the forest, and are used instead of modern health centers that are (c) too far away, (d) too slow to treat people, and (e) too expensive
Honey	illegal for majority; 217 people authorized to harvest from beehives (12.1% of total sample)	15.0 (7.2, 0.038)	obtaining honey from hives kept in the park	0.342 (a) 0.276 (b) 0.236 (c)	collected to (a) sell, (b) eat, and (c) use as medicine
Basketry materials	illegal for majority; 336 authorized to harvest *Smilax anceps* only	NA	NA	0.322 (a) 0.285 (b)	*Smilax anceps* and *Loeseneriella apocynoides* collected to make baskets and trays (a) to sell and (b) for household purposes
Building poles	illegal for all	13.6 (6.7, 0.044)	using poles from the park for construction	0.110	collected for household use

a Proportion of households in the sample (*n* = 365) estimated to have used the resource in the 12 months prior to interview, with the exception of medicinal plants, for which 6 months was used. The *p* values are probability that the prevalence of resource use is >0 (i.e., that the null hypothesis is rejected).

b Range 0–1 (1, motivation ranked first in all lists; <1, motivation ranked lower or included in fewer lists).

c Matching letters indicate corresponding explanation of the salience score for that resource.

### Resource Users

**Figure 1:**
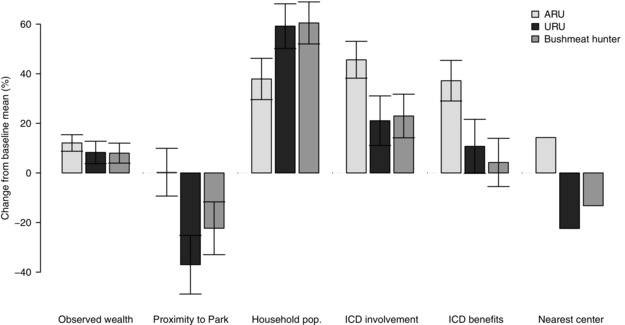
Percent variation in socioeconomic characteristics of resource user groups relative to the baseline sample mean (*p* values in Supporting Information; ARU, authorized resource user, member of the Multiple Use Program permitted to harvest one or more of honey, medicinal plants, and basketry materials from designated areas of the national park; URU, unauthorized resource user, arrested for unauthorized activity in the national park from August 2012 to July 2013; bushmeat hunter, URUs arrested for bushmeat hunting in the national park from January 2011 to July 2013; observed wealth, household wealth score assigned based on observation; household pop, number of people in the household; ICD involvement, perceived involvement in the design and implementation of integrated and conservation development [ICD] projects; ICD benefits, total number of ICD projects respondents perceived themselves to have benefitted from; nearest center, proportion of households living within 1 hour of a trading center).

Relative to the baseline sample (assumed non‐resource users), ARUs had higher observed wealth scores and were more likely to live within an hour of a trading center. The URUs were more likely to live over an hour from the nearest trading center and closer to Bwindi's boundary. Both ARUs and URUs had larger households than the baseline sample. Both ARUs and URUs felt more involved with the development and implementation of ICD projects, but only ARUs thought they benefitted from significantly more projects than the baseline population (Fig. 1).

Not all types of URU had the same socioeconomic profile. People apprehended for bushmeat hunting (*n* = 46) lived closer to Bwindi's boundary and came from larger households relative to the baseline sample but had higher observed wealth scores and were no more or less likely to live near or far from trading centers. The UCT data gave an alternative profile of general bushmeat consumers (as opposed to only apprehended hunters). Similar to the arrested hunters, bushmeat consumers lived close to Bwindi's boundary and thought they benefitted from the MUP, although they were highly unlikely to be ARUs. They were more likely than others to come from single households, rate their lives average or somewhat bad as opposed to worst, and have less education than average (Table 2). Firewood users tended to live over an hour from the nearest center and had more education than average (Table 3).

**Table 2:** Estimates and relative importance of variables included in the averaged top model for the unmatched count technique profile of people who consumed bushmeat in the year prior to survey

					It is likely that people who
					have consumed bushmeat in
Variable	Response	Estimate^a^	SE	RVI^b^	the year prior to survey…
ARU^c^	yes	−0.652	0.218	1.00	are not ARUs
Marital status	single	0.619	0.216	0.99	are from single parent households
Benefit from MUP^d^	yes	0.424	0.172	0.95	have benefitted from MUP
Well‐being^e^	somewhat bad	0.030	0.223	0.95	rate their lives average or somewhat bad
	worst	−0.644	0.319		
Education	4 years or more	−0.309	0.174	0.45	have 3 or fewer years of formal education

a Change in probability that a household consumed bushmeat in the year prior to survey relative to the sample average.

b Relative variable importance: proportion of top models the variable is included in. Only variables with RVI >0.4 are included in the table (see Supporting Information for details).

c Authorized resource user.

d Multiple use program.

e Respondents were asked which word or phrase best represented their lives: *worst*, *somewhat bad, average, fine*, *best*. No one answered *fine* or *best*.

**Table 3:** Estimates and relative importance of variables included in the averaged top model for the unmatched count technique profile of people who collected firewood from the national park in the year prior to survey

					It is likely that people who
					collected firewood from the
Variable	Response	Estimate^a^	SE	RVI^b^	Park in the year prior to survey…
Nearest center	under 1 hour	−0.537	0.221	0.84	live over an hour from the nearest trading center
Education	4 or more years	0.321	0.173	0.63	have at least 4 years of education

a Change in probability that a household used firewood from the national park in the year prior to survey relative to the sample average.

b Relative variable importance: the proportion of top models the variable is included in. Only variables with RVI >0.4 are included in the table, (see Supporting Information for details).

### Reasons for Resource Use

Focus groups reported 3 categories of motivations for resource use: poverty, resentment, and cultural beliefs. Poverty meant that resources were not available or were in short supply outside Bwindi and that people lacked money with which to purchase alternatives. Resources were therefore sought primarily for household consumption; surplus was sold to generate low levels of supplementary income (Table 1). Both stretcher groups and Reformed Poachers Associations stated that bushmeat is hunted primarily for private consumption in households where food, particularly protein, is lacking and is used to treat severe childhood malnutrition. Bushmeat is traded locally when there is excess and profits are used to pay school fees, for example. The Batwa believe bushmeat is medicinal and passes on traditional knowledge to children who consume it. Local people also harvested bamboo for hoe handles and fishing. Only timber and gold were extracted for income, but relatively few people engaged in this activity (Supporting Information).

Resentment toward Bwindi was almost as strong a motivation as poverty. Local people considered Bwindi contributed to or failed to alleviate poverty as intended due to crop raiding, inequity of revenue sharing, and lack of employment. Anger about crop raiding and the lack of support from park authorities was the motivation with the fifth highest salience score out of 59 (S = 0.378) and was discussed passionately and at length in all focus groups. Crop raiding results in food scarcity for the household and a loss of income. There is no direct financial compensation for crop raiding and, of the household survey respondents aware of crop raiding interventions (*n* = 341 out of 365), most (59.5%) thought that they received no benefit or that interventions did not change their situation. People affected by crop raiding therefore perceived little problem with compensating themselves with resources from the park.

Anger at the lack of Park‐related employment for local people had similar salience to motivations for harvesting honey and basketry materials. Local people perceived that, despite applying for jobs and performing well at interview, jobs went to people from distant areas who were related to park employees.

Local people thought management and distribution of revenue was corrupt and that over half of the money was “lost” as it passed through layers of government. Focus group participants thought revenue tended to be shared with wealthy people living in population centers and in positions of power. Anger at the corruption and inequity of revenue sharing as a motivation for unauthorized resource use ranked similarly to lack of employment. As one focus group participant stated, “People are angered by the revenue sharing of giving goats. Those who are benefitting by receiving goats are those who are not living near the park. People near the park (like us) are denied goats, so we are angry and go to the park and poach.”

**Figure 2:**
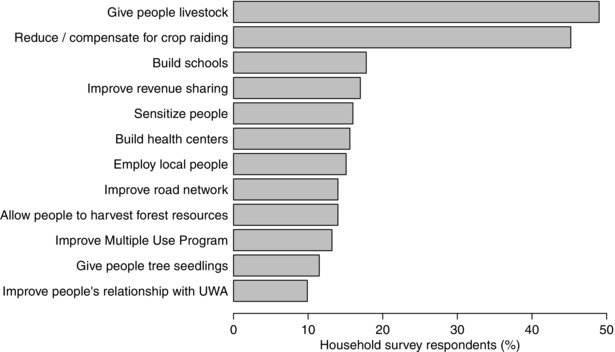
The percentage of survey respondents who said they would undertake the most commonly mentioned activities if they were the national park manager (*n* = 365).

The importance of crop raiding, inequity of revenue sharing, and lack of employment to local people was also apparent from the household survey. When asked what they would do if they were park manager, 45.2% responded they would take action against crop raiding, second only to providing livestock to more people and in an equitable manner (49.0%). Seventeen percent and 15.1% said they would improve the process of revenue sharing and employ local people, respectively (Fig. 2).

A relatively minor motivation of unauthorized resource use was cultural belief. For example, herbal medicine is sought because it is trusted more than modern medicine. A commonly mentioned but lowly ranked motivation for entering the Park was being driven into the forest by evil spirits (*S* = 0.152).

### Deterrents Against Resource Use

**Figure 3:**
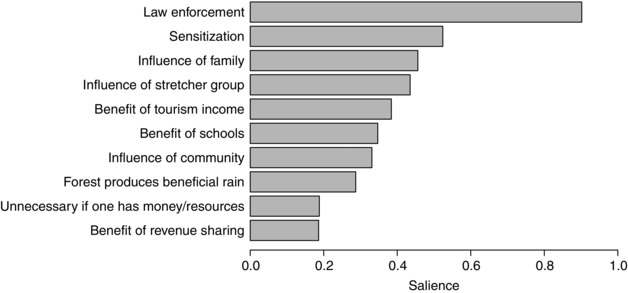
Salience score of the top deterrents to unauthorized activity in the national park as ranked by 17 focus groups from local community institutions known as stretcher groups (score of 1, deterrent was ranked first by all focus groups; score <1, deterrent ranked lower or not ranked in every group).

The deterrent with the highest overall salience was law enforcement, followed by sensitization regarding the environmental and economic importance of Bwindi, social influence, and benefits from Bwindi (Fig. 3). Additional reasons for not entering Bwindi included not needing the resources or the money generated through their sale. Other minor reasons included religion, fear of the forest, and lack of energy (Supporting Information).

Despite being ranked lower than law enforcement overall, ICD benefits ranked higher than law enforcement as a deterrent to unauthorized resource use in communities that perceived themselves to have benefitted substantially and fairly from Bwindi, although there were insufficient data to confirm this quantitatively. For example, in a community adjacent to Bwindi's tourism center and host to the Bwindi Village Walk, a popular tourist venture, law enforcement was ranked as the least important deterrent. This focus group listed 12 deterrents, 9 of which were benefits they perceived themselves to receive from Bwindi, and ranked law enforcement last.

## Discussion

Ours is the first study to comprehensively characterize the profiles and motivations of authorized and unauthorized resource users through the application of triangulated methods to strengthen the robustness of the results. We found high levels of unauthorized consumption of bushmeat and firewood; 26% (SE 8) and 20% (SE 9) respectively of surveyed households used these resources in the year prior to survey. Use of medicinal plants (16%) was higher than would be expected from the low proportion of authorized harvesters (4% of sample). We found that profiles and motivations of resource users were complex and diverse and that, compared to the rest of the population, ARUs were relatively less poor and URUs were often marginalized across a range of dimensions.

Our findings suggest a shift from the early 1990s, when Tukahirwa and Pomeroy (1993) surveyed people about their use of forest resources before park gazettement. As a game reserve, harvest of certain resources was allowed with permits. Bushmeat hunting was forbidden but went largely unchecked (Namara 2000). Tukahirwa and Pomeroy (1993) reported 40–60% of households harvesting various timber products and plants for medicine and basketry, but only 10% of their sample reported hunting bushmeat. Only 1% of people admitted to continuing to take firewood, and none admitted to hunting after gazettement. However, Tukahirwa and Pomeroy (1993) used direct questioning to ascertain resource use, and people are likely to have felt uncomfortable responding truthfully, even when asked about past activity. Baker (2004) found that the number of snares found by rangers in the forest remained relatively constant over the period prior to and following gazettement. This suggests that our higher estimate of bushmeat use could be a consequence of our use of an indirect questioning method to obtain more reliable estimates of a sensitive behavior.

We found that ARUs displayed characteristics typical of comparatively wealthy households, such as living within an hour of trading centers and not close to Bwindi's boundary, and were significantly less poor than the general sample. This may be as a result of their participation in the MUP but also could be because the MUP tends to benefit already wealthier members of the community (Blomley et al. 2010; Shirkhorshidi 2013). This fits with other literature which suggests that participation in conservation interventions such as payment for environmental services schemes is often dominated by households who are already better off and so better able to access the opportunities provided (Sommerville et al. 2010; Clements & Milner‐Gulland 2015). Similarly, previous studies show that tourism‐related employment at Bwindi is more likely to benefit wealthier, well‐educated males living close to the park gate (Sandbrook & Adams 2012).

Focus groups suggested that unauthorized harvesting is driven by the inability to afford or access resources outside Bwindi. The larger household size of both ARUs and URUs may be because they have more people with which to diversify livelihood strategies and can invest in less profitable or higher risk activities, as is commonly seen in natural‐resource dependent societies (Ellis 2000). Shirkhorshidi (2013) found that inactive ARUs at Bwindi came from smaller households and that one reason for inactivity was that the MUP was not meeting their economic livelihood needs, which supports our suggestion that natural resource use is more possible for households with sufficient labor.

The UCT bushmeat profile showed strongly that ARUs are very unlikely to consume bushmeat, despite a small proportion of ARUs having been apprehended for hunting in Bwindi. Despite opposition to the MUP when it was first established (Blomley 2003), out of concern that it would be used as a cover for unauthorized resource use, we found no evidence suggesting this is currently the case. This could be because ARUs lose their right to access resources if caught engaging in unauthorized activity and do not perceive it to be worth the risk.

Our results suggest that unauthorized harvesting of resources is motivated by both poverty and resentment. Instances of retaliatory killings of protected carnivores responsible for livestock predation are not uncommon (e.g., Kissui 2008; Hazzah et al. 2009), including in Uganda (Omoya & Plumptre 2011). However, harvesting resources inside a protected area because of resentment appears to be rarely reported in the literature (but see Badola 1998).

Resentment toward Bwindi due to crop raiding, perceived inequity of revenue sharing, or lack of employment may arise regardless of socioeconomic status or positive attitudes generated by ICD projects. However, it is likely that resentment and poverty are linked. First, crop raiding has a greater impact on the attitudes of the poor toward the conservation of Bwindi (Blomley et al. 2010) because they are less able to cope with the consequences. Second, the same issues that drive resentment exacerbate poverty; crop raiding makes people poorer, and inequitable revenue sharing and lack of employment fail to contribute to its alleviation. Finally, previous studies show wealthier households have positive attitudes toward Bwindi's conservation regardless of the benefits they receive (or do not receive) through ICD (Blomley et al. 2010).

We suggest targeting ICD projects specifically toward the poorest people living close to Bwindi's boundary who are therefore more likely to be URUs. Because most resources were harvested for household use rather than for sale, ICD projects could target poverty alleviation through provision of alternative sources of meat, fuel, and medicinal plants, which are currently harvested from the Park. Current and future ICD projects should be reviewed and managed in a transparent and equitable manner to avoid resentment due to their perceived mismanagement, particularly because our findings regarding the perceived inequity of revenue sharing are in line with the results of other studies around Bwindi (e.g., Ahebwa et al. 2012; Tumusiime & Vedeld 2012). In 2014, the Uganda Wildlife Authority increased the gorilla levy to be shared with local communities from $5 to $10 per permit. Members of Uganda's Poverty and Conservation Learning Group advocated for this change based on the findings reported here. Finally, crop raiding remains a significant problem at Bwindi, despite years of prevention and mitigation strategies (Aharikundira & Tweheyo 2011), suggesting further engagement with local people is required.

Understanding the profiles and motivations of resource users is vital to targeting conservation interventions effectively. To do so is difficult due to the sensitive nature of the behavior under investigation. Our research illustrates that a mixed methods approach, combining indirect and direct questions and individual interviews with focus group discussions, is highly conducive to understanding the complex drivers of unauthorized resource use, allowing triangulation of findings and different views of the same story. We also demonstrated the effectiveness of using existing records of both authorized and unauthorized resource users combined with a survey of the general population to establish differences between groups. Profiling known resource users allowed us to cross‐validate the results of the UCT survey and focus group discussions. By taking advantage of the unusual opportunity for cross‐validation provided by the URU and ARU data sets, we were able to show that the results from the UCT study were congruent with information about known resource users and insights from focus groups. This suggests that this quick, simple, and relatively unthreatening method can yield robust information about unauthorized resource users. Our findings highlight the marginalized nature of URUs at Bwindi, both geographically and in terms of livelihood options, their failure to benefit from current conservation interventions, and point to a way forward for conservationists to better engage with this group and overcome limitations to equitable ICD in the future.

## Supporting information

Details of the survey methods, including how ethical issues were addressed, the UTC (Appendix S1), and of socioeconomic variables, UCT models, and saliences of all motivations and deterrents (Appendix S2) are available online. The authors are solely responsible for the content and functionality of these materials. Queries (other than absence of the material) should be directed to the corresponding author.
Figure S1. Sample Unmatched Count Technique cards, showing the control card on the left and the treatment card on the right, including the sensitive item, in this case bushmeat.
Table S1. Changes in wealth and education according to education and proximity to Bwindi Impenetrable National Park, roads and trading centers.
Table S2. Variation in socioeconomic characteristics of resource user groups compared to the baseline sample mean.
Table S3. Set of models selected based on AICc for bushmeat consumption.
Table S4. Set of models selected based on AICc for firewood collection from the park.
Table S5. Motivations for resource use, as ranked by 17 focus groups.
Table S6. Deterrents against resource use, as ranked by 17 focus groups.
