# Supplementary material for: Profiling unauthorized natural resource users for better targeting of conservation interventions
Source: Conserv Biol. 2015 Aug 3;29(6):1636–46. doi: 10.1111/cobi.12575 (PMC4975694; doi:10.1111/cobi.12575)

Profiling unauthorized natural resource users for better targeting of conservation interventions

**Supporting Information**

**Appendix S1** Details of household survey, Unmatched Count Technique, focus group discussion methodology and ethics review procedure

*Household survey*

The household survey was conducted with 365 respondents between April and August 2013. Local field assistants, who had been trained in social survey techniques, held interviews in the local language of Rukiga. Permission was sought from local village leaders before any interviews began. Before the survey, each participant was informed of the purpose of the research in the context of the “Research to Policy: Building Capacity for Conservation through Poverty Alleviation in Uganda” project. Only senior members of the research team were aware of why each person was being interviewed, and all field staff were fully briefed on the importance of confidentiality. No respondent was told why they specifically had been selected for interview, as is the case with most social surveys, regardless of the sampling method. This means that unauthorized resource users were not told that they were being interviewed as people who had previously been arrested. All participants were assured of the confidentiality of the study. Data was entered into a MS Access database designed for the project. All identifying information was removed before data was entered into the database. Respondents were compensated for their time given to interviews in line with Institute of Tropical Forest Conservation (ITFC) protocol.

The observed household wealth score was based on the structure and material of the participants’ primary dwelling. The features of a dwelling associated with wealth were identified during a focus group with local people. The materials of the walls, wall coating and roof of the main house, kitchen and latrine were observed and scored according to the materials they were composed of and how permanent they were. In addition, households scored higher if they possessed a water tank. The maximum score was 31 (Harrison 2013).

To obtain information about a household’s involvement and benefit from Integrated Conservation and Development (ICD) projects, participants were first asked to list all projects that they were aware of. For each of these projects, the participant was asked if they felt that they had benefited from that project. We did not quantify the benefit, or confirm the material benefit they might have received from secondary sources, as we felt it more important to know whether or not a participant perceived themselves to have benefited, whether directly or indirectly.

For each ICD project a participant was aware of, we also asked how involved they felt that they had been in the design and implementation of the project from the categories “Not at all”, “A little”, “Somewhat” or “A lot”. The participants perceived involvement in each ICD project was scored 0-3, and a total involvement score calculated as the sum of each individual project involvement score. A higher score therefore denotes feeling more involved in more projects, whilst a low score could mean that a person felt a little involved in many projects, or very involved in just a few.

*Unmatched Count Technique*

Whilst indirect questioning requires a large sample size and a level of expertise in survey design and implementation, it is one of the most reliable methods of estimating levels of illegal activity. It allows the interviewee to give an honest response, without revealing to the researcher whether or not they engage in a sensitive behavior. The Unmatched Count Technique (UCT) is one such method, and has been found to give significantly higher estimates of illegal activities than direct questioning (Nuno et al. 2013). The limitations of UCT, however, are that it requires a large sample size, and is not effective at estimating the prevalence of behaviors involving a small proportion of the population.

The UCT questions were designed to establish the prevalence of extraction of medicinal plants, honey, firewood, bushmeat and building poles. For each resource, interviewees were offered two cards facing down (one control and one treatment) and asked to pick one at random without seeing what each displayed. Interviewees were asked “How many of the places shown on the card have you obtained [medical treatment / honey / energy for cooking / meat / building materials] from in the past year?”

The control cards showed four photographs of potential sources of each resource, including one that everybody was expected to include and one that almost nobody would use, to avoid the loss of confidentiality that would occur if either all or none of the items in the photographs were counted. The treatment cards showed the same four photographs with an additional photograph of the resource extracted from the Park. The photograph of the Park was randomly positioned on each card. In addition to the photographs, the cards had an explanation of each photograph written in both English and the local language, Rukiga, and the photographs were explained to each respondent by the field assistants before they answered, to account for illiteracy (Figure S1).

To prepare the respondent for the UCT questions and identify any misunderstandings in the methodology, a trial question about crop raiding was asked before questions about forest resources. After the method was explained to the interviewee, they were asked to select a card then answer the question “How many of these animals have your crops been raided by in the past year?”

Items for inclusion on all cards were selected by MH and Stephen Asuma, Uganda Country Co-ordinator for the International Gorilla Conservation Program, who has worked closely with communities around Bwindi for the past 15 years. He could therefore advise the alternative sources of forest resources that local people were likely (and unlikely) to utilize. Before implementation, the items were reviewed and revised where necessary by the field team.

*Focus group discussions*

Stretcher groups in southwest Uganda are informal governance institutions, usually comprised of members of the same clan living in the same locality (Katabarwa 1999). The original purpose of the stretcher group is to provide assistance when a member is sick, by carrying them to hospital or the nearest road on a traditional stretcher woven from plant materials gathered from the forest. If somebody dies, the group provides firewood for the funeral pyre, money to assist with costs, and food to cater for the visiting mourners. Some stretcher groups have expanded their activities to include income-generating projects, small loan schemes and other developmental activities, (C. Byaruhanga, personal communication).

Stretcher groups were identified as suitable for focus group discussions because they have constitutions and by-laws with which to govern their members. They regularly review their moral code for their community and provide guidance and advice as to how to behave. Some groups impose fines and punishments on members who disobey (Katabarwa 1999), so were considered to be well placed to give information regarding unauthorized activity.

Stretcher groups were selected in parishes and villages where a high number of household surveys were undertaken, in case comparisons could be made between the qualitative information collected in the discussions and the quantitative data derived from the interviews. Parishes were also selected to provide variation across the following variables: presence/absence of habituated gorillas for tourism, participation in the Multiple Use Program (MUP), and number of Authorized Resource Users (ARUs) and Unauthorized Resource Users (URUs). In total, 17 focus groups with stretcher groups were held in eight parishes (Harrison 2013).

The village chairman was contacted to identify the names and contact details of the stretcher groups in each selected village, from which one was randomly selected. In areas where there were Batwa communities, focus groups were held with both a Batwa and a Bakiga stretcher group from the same locality, to allow for comparisons where all other factors are relatively constant.

At the time of survey, there were three Reformed Poachers Associations (RPAs) around Bwindi, in the parishes of Mpungu, Kiyebe and Rubuguri (Harrison 2013). Focus group discussions were held with members of each one. The RPA’s were established with the assistance of a Community Conservation Ranger (CCR) from the Uganda Wildlife Authority, with a poaching equipment amnesty following a short period of sensitization. The CCR provided the field team with the contact details of each group’s Chairman.

The stretcher group or RPA chairman was phoned in advance and asked to assemble a group of between six and 10 people ranging in age and gender from his group. The location was arranged in advance to be somewhere enclosed, private and relatively quiet, to prevent distractions or interruptions. The chairman was not informed that a *mzungu* (white person) would be leading the discussion, just that researchers from ITFC, which is relatively well-known around the Park, would be present, so as not to raise the expectations of the participants or gather more members than were required.

Discussions were led by MH and facilitated by a field assistant from ITFC fluent in both English and Rukiga and trained in social research techniques. Each focus group was compensated for their time and travel expenses in accordance with ITFC protocol. Audio recordings were made of focus group discussions with the participants’ permission. Additional translations were made from the recordings with the assistance of the field assistant.

Groups were assured that the purpose of the discussions was not to incriminate anybody, but to help to find solutions for both people and the Park. MH explained that often people behave illegally because they have problems, either with the Park or in their own lives, and that if we understand the problems then we have a greater chance of solving them, so that people have better lives and so that the forest is better protected and lasts long into the future.

*Ethics review*

The ethics of using known offenders and the risk of incriminating individuals were discussed and reviewed at length before the research design was finalized. This approach enabled a far greater insight into the profiles of unauthorized resource users than would otherwise have been possible, and was therefore, we feel, justified according to the arguments made by Miller (1995). However, the ethics of doing so were heavily scrutinized by each project partner and our project stakeholders as follows.

This research was conducted as part of the lead author’s MSc project, and as such was approved by the MSc Course Directors, based on submission of an Ethics Statement, review by the Directors and subsequent submission of a revised Ethics Statement. Imperial College’s research ethics committee does not cover research done as part of a taught course. The research was also reviewed by the lead partner in the overall research project, the International Institute for Environment and Development. Their procedure involves circulation of the protocols to expert colleagues, and scrutiny against IIED's ethical principles (<http://www.iied.org/our-research-striving-towards-excellence>). The research design went through several reviews before being accepted, and this resulted in our research emphasis on ‘unauthorized’ resource use, rather than illegal activities, and (as part of the wider project) on the project team working to change how such use is perceived, namely as an indicator of livelihood needs of local people rather than as ‘illegal activities’. In addition, the ethics of the study, such as including known offenders in a survey, was discussed at length at a project planning workshop attended by all partner organisations, members of the Uganda Poverty and Conservation Learning Group and the Uganda Wildlife Authority (UWA). Ethical protocols of the research were then reviewed at the Interim Research Workshop held by the project team with UWA at Bwindi. Project partner the Institute for Tropical Forest Conservation has an MoU with UWA, allowing them to request access to UWA data for research purposes, and permission was granted to the project team from UWA, under this MoU, to use the lists of known offenders on the understanding that strict protocols regarding their anonymity would be enforced, whereby no one but the senior members of the research team would know their names, a condition which was adhered to throughout.

**Literature Cited**

Harrison, M., 2013. Establishing profiles and motivations of resource users at Bwindi Impenetrable National Park, Uganda. MSc thesis. Imperial College London. Available at: http://www.iccs.org.uk/wp-content/thesis/consci/2013/Harrison.pdf [Accessed November 10, 2013]

Katabarwa, M., 1999. Modern health services versus traditional engozi system in Uganda. The Lancet **354**: 343.

Miller, M., 1995. Covert participant observation: reconsidering the least used method. Journal of Contemporary Criminal Justice **11**: 97–105

Nuno, A., Bunnefeld, N., Naiman, L., Milner-Gulland, E.J., 2013. A Novel Approach to Assessing the Prevalence and Drivers of Illegal Bushmeat Hunting in the Serengeti. Conservation Biology **27**: 1355–1365.

**Appendix S2** Socioeconomic variables, Unmatched Count Technique models, and saliences of all motivations and deterrents

**Table S1**. Changes in wealth and education according to education and proximity to Bwindi Impenetrable National Park, roads and trading centers. The observed wealth score ranges between 4 and 22, with a mean of 13.4.

|  | Observed wealth score | | | Education (years) | | |
| --- | --- | --- | --- | --- | --- | --- |
|  | Est. | SE | p-value | Est. | SE | p-value |
| Education (< 4 years) | -1.62 | 0.23 | <0.001 | NA | NA | NA |
| Proximity to Park (< 0.5 km) | -2.00 | 0.39 | <0.001 | -1.39 | 0.41 | 0.002 |
| Nearest road (> 1 hour) | -1.46 | 0.36 | <0.001 | -0.98 | 0.38 | 0.021 |
| Nearest center (> 1 hour) | -1.26 | 0.38 | 0.003 | -0.62 | 0.43 | 0.157 |

**Table S2**. Variation in socioeconomic characteristics of resource user groups compared to the baseline sample mean.

| Variable | Type of resource user^a^ | Change^b^ | SE | p-value | Sig^c^ |
| --- | --- | --- | --- | --- | --- |
| Observed wealth score | ARU | 12.09 | 3.33 | 0.0002 | *** |
| Observed wealth score | URU | 8.28 | 4.5 | 0.1155 | ns |
| Observed wealth score | Bushmeat hunter | 7.98 | 4.03 | 0.0484 | * |
|  |  |  |  |  |  |
| Proximity to Park | ARU | 0.3 | 9.61 | 0.969 | ns |
| Proximity to Park | URU | -37.01 | 11.83 | 0.0008 | *** |
| Proximity to Park | Bushmeat hunter | -22.3 | 10.64 | 0.0375 | * |
|  |  |  |  |  |  |
| Household Size | ARU | 37.9 | 8.32 | <0.0001 | *** |
| Household Size | URU | 59.2 | 9.01 | <0.0001 | *** |
| Household Size | Bushmeat hunter | 60.5 | 8.48 | <0.0001 | *** |
|  |  |  |  |  |  |
| Involvement in ICD | ARU | 45.64 | 7.43 | <0.0001 | *** |
| Involvement in ICD | URU | 21.08 | 10 | 0.0355 | * |
| Involvement in ICD | Bushmeat hunter | 22.97 | 8.79 | 0.0096 | ** |
|  |  |  |  |  |  |
| ICD benefits | ARU | 37.2 | 8.19 | <0.0001 | *** |
| ICD benefits | URU | 10.72 | 10.9 | 0.326 | ns |
| ICD benefits | Bushmeat hunter | 4.24 | 9.72 | 0.664 | ns |
|  |  |  |  |  |  |
| Trading centre <1hr | ARU | 14.3 | - | 0.04 | * |
| Trading centre <1hr | URU | -22.4 | - | 0.017 | * |
| Trading centre <1hr | Bushmeat hunter | -13.2 | - | 0.1472 | ns |

^a^ ARU: Authorized Resource User; URU: Unauthorized resource user; Bushmeat hunter: Subset of URUs arrested for bushmeat hunting in the national park between January 2011 and July 2013.

^b^ Change: Percentage change of resource user mean from baseline mean

^c^ Significance levels: ns = not significant (>0.1), . = 0.1, * = 0.05, ** = 0.01, *** = 0.001

**Table S3** Set of models selected based on AICc for bushmeat consumption. x indicates that the variable was included in the model. The degrees of freedom (df), the log-likelihood (log(L)), the information criterion value (AICc), the AICc difference (ΔAICc) and AICc weight are given for each model. The Relative Variable Importance (RVI) is given at the end. Variables removed from the model during simplification were district, water source (protected or unprotected), sanitation level (access to hand-washing facilities, a drying rack and kitchen with a smoke escape outlet), and whether or not the respondent perceived themselves to have benefitted from a tree-planting or livestock provision project.

|  | card | aru | aru* | wb | hh | hh* | mup | mup* | wb* | edu | edu* | neigh | dist | meet | neigh* | dist* | meet* | cent | cent* | df | log(L) | AICc | ΔAICc | weight |
| --- | --- | --- | --- | --- | --- | --- | --- | --- | --- | --- | --- | --- | --- | --- | --- | --- | --- | --- | --- | --- | --- | --- | --- | --- |
| 1 | x | x | x | x | x | x | x | x | x | x | x |  |  |  |  |  |  |  |  | 15 | -390.61 | 812.64 | 0.00 | 0.06 |
| 2 | x | x | x | x | x | x | x | x | x |  |  |  |  |  |  |  |  |  |  | 13 | -393.18 | 813.43 | 0.79 | 0.04 |
| 3 | x | x | x | x | x | x | x |  | x |  |  | x |  |  | x |  |  |  |  | 15 | -391.11 | 813.65 | 1.01 | 0.04 |
| 4 | x | x | x | x | x | x | x | x | x | x |  |  |  |  |  |  |  |  |  | 14 | -392.11 | 814.01 | 1.37 | 0.03 |
| 5 | x | x | x | x | x | x | x | x | x | x | x |  | x |  |  | x |  |  |  | 17 | -392.39 | 814.14 | 1.50 | 0.03 |
| 6 | x | x | x | x | x | x | x | x | x | x | x | x |  |  |  |  |  |  |  | 16 | -389.16 | 814.18 | 1.54 | 0.03 |
| 7 | x | x | x | x | x | x | x | x | x | x | x | x |  |  | x |  |  |  |  | 17 | -390.28 | 814.23 | 1.59 | 0.02 |
| 8 | x | x | x | x | x | x | x | x | x | x | x |  | x | x |  | x | x |  |  | 19 | -389.20 | 814.42 | 1.78 | 0.02 |
| 9 | x | x | x | x | x | x | x | x | x |  |  | x |  |  |  |  |  |  |  | 14 | -387.07 | 814.58 | 1.94 | 0.02 |
| 10 | x | x | x | x | x | x | x | x | x | x |  | x |  |  | x |  |  |  |  | 16 | -392.67 | 814.60 | 1.96 | 0.02 |
| 11 | x | x | x | x | x | x | x | x | x | x | x |  |  | x |  |  |  |  |  | 16 | -390.49 | 814.61 | 1.97 | 0.02 |
| 12 | x | x | x | x | x | x | x | x | x | x | x |  | x |  |  |  |  |  |  | 16 | -390.50 | 814.65 | 2.01 | 0.02 |
| 13 | x | x | x | x | x | x | x | x | x | x | x |  |  |  |  |  |  | x |  | 16 | -390.54 | 814.69 | 2.06 | 0.02 |
| 14 | x | x | x | x | x | x | x | x | x | x | x |  |  | x |  |  | x |  |  | 17 | -389.60 | 815.03 | 2.39 | 0.02 |
| 15 | x | x | x | x | x | x | x | x | x |  |  |  | x |  |  | x |  |  |  | 15 | -391.92 | 815.25 | 2.62 | 0.02 |
| 16 | x | x | x | x | x | x | x | x |  |  |  |  |  |  |  |  |  |  |  | 11 | -396.24 | 815.26 | 2.62 | 0.02 |
| 17 | x | x | x | x | x | x | x | x | x |  |  | x | x |  | x | x |  |  |  | 17 | -389.72 | 815.26 | 2.62 | 0.02 |
| 18 | x | x | x | x | x | x |  |  | x | x | x |  |  |  |  |  |  |  |  | 13 | -394.13 | 815.32 | 2.69 | 0.02 |
| 19 | x | x | x | x | x | x | x | x | x | x | x |  |  |  |  |  |  | x | x | 17 | -389.75 | 815.33 | 2.69 | 0.02 |
| 20 | x | x | x | x | x | x | x | x | x |  |  |  |  | x |  |  |  |  |  | 14 | -393.06 | 815.37 | 2.73 | 0.02 |
| 21 | x | x | x | x | x | x |  |  | x |  |  |  |  |  |  |  |  |  |  | 11 | -396.32 | 815.41 | 2.77 | 0.01 |
| 22 | x | x | x | x | x | x | x | x | x |  |  |  |  |  |  |  |  | x |  | 14 | -393.09 | 815.42 | 2.79 | 0.01 |
| 23 | x | x | x | x | x | x | x | x | x |  |  |  | x | x |  | x | x |  |  | 17 | -389.80 | 815.43 | 2.79 | 0.01 |
| 24 | x | x | x | x | x | x | x | x | x |  |  |  | x |  |  |  |  |  |  | 14 | -393.13 | 815.50 | 2.86 | 0.01 |
|  | card | aru | aru* | wb | hh | hh* | mup | mup* | wb* | edu | edu* | neigh | dist | meet | neigh* | dist* | meet* | cent | cent* | df | log(L) | AICc | ΔAICc | weight |
| 25 | x | x | x | x | x | x | x | x | x | x |  | x |  |  |  |  |  |  |  | 15 | -392.07 | 815.55 | 2.91 | 0.01 |
| 26 | x | x | x | x | x | x | x | x | x | x | x | x | x |  | x | x |  | x |  | 19 | -387.66 | 815.59 | 2.96 | 0.01 |
| 27 | x | x | x | x | x | x | x | x | x |  |  |  |  | x |  |  | x |  |  | 15 | -392.10 | 815.62 | 2.98 | 0.01 |
| 28 | x | x | x | x | x | x | x | x | x | x |  |  | x |  |  | x |  |  |  | 16 | -391.01 | 815.63 | 2.99 | 0.01 |
| 29 | x | x | x | x | x | x | x | x | x |  |  |  |  |  |  |  |  | x | x | 15 | -392.11 | 815.64 | 3.01 | 0.01 |
| 30 | x | x | x | x | x | x | x | x | x |  |  | x |  | x | x |  |  |  |  | 16 | -391.04 | 815.69 | 3.05 | 0.01 |
| 31 | x | x | x | x | x | x | x | x | x | x |  |  | x | x |  | x | x |  |  | 18 | -388.83 | 815.70 | 3.06 | 0.01 |
| 32 | x | x | x | x | x | x | x | x | x |  |  | x | x | x | x | x | x |  |  | 19 | -387.73 | 815.74 | 3.11 | 0.01 |
| 33 | x | x | x | x | x | x | x | x | x |  |  | x | x |  | x |  |  |  |  | 16 | -391.07 | 815.75 | 3.11 | 0.01 |
| 34 | x | x | x | x | x | x | x | x | x |  |  | x |  |  | x |  |  | x |  | 16 | -391.08 | 815.77 | 3.13 | 0.01 |
| 35 | x | x | x | x | x | x | x | x | x | x | x | x | x |  |  | x |  |  |  | 18 | -388.87 | 815.78 | 3.14 | 0.01 |
| 36 | x | x | x | x | x | x | x | x | x | x | x |  | x | x |  | x |  |  |  | 18 | -388.90 | 815.84 | 3.20 | 0.01 |
| 37 | x | x | x | x | x | x |  |  | x | x |  |  |  |  |  |  |  |  |  | 12 | -395.52 | 815.95 | 3.32 | 0.01 |
| 38 | x | x | x | x | x | x | x | x | x | x |  | x | x |  | x | x |  |  |  | 18 | -388.98 | 816.00 | 3.36 | 0.01 |
| 39 | x | x | x | x | x | x | x | x | x | x |  |  |  | x |  |  |  |  |  | 15 | -392.30 | 816.01 | 3.38 | 0.01 |
| 40 | x | x | x | x |  |  | x | x | x | x | x |  |  |  |  |  |  |  |  | 13 | -394.49 | 816.04 | 3.40 | 0.01 |
| 41 | x | x | x | x | x | x | x | x | x |  |  | x |  |  | x |  |  | x | x | 17 | -390.11 | 816.05 | 3.41 | 0.01 |
| 42 | x | x | x | x | x | x | x | x | x | x |  |  | x |  |  |  |  |  |  | 15 | -392.33 | 816.08 | 3.45 | 0.01 |
| 43 | x | x | x | x | x | x | x | x | x | x |  |  |  |  |  |  |  | x |  | 15 | -392.34 | 816.11 | 3.47 | 0.01 |
| 44 | x | x | x | x | x | x | x | x | x |  |  | x |  | x | x |  | x |  |  | 17 | -390.14 | 816.11 | 3.47 | 0.01 |
| 45 | x | x | x | x | x | x | x | x | x | x | x | x | x | x | x | x | x |  |  | 21 | -385.67 | 816.11 | 3.48 | 0.01 |
| 46 | x | x | x | x | x | x | x | x |  |  |  | x |  |  |  |  |  |  |  | 12 | -395.60 | 816.12 | 3.48 | 0.01 |
| 47 | x | x | x | x | x | x | x | x | x | x | x | x | x |  |  |  |  |  |  | 17 | -390.17 | 816.16 | 3.52 | 0.01 |
| 48 | x | x | x | x | x | x | x | x |  |  |  | x |  |  | x |  |  |  |  | 13 | -394.55 | 816.17 | 3.53 | 0.01 |
| 49 | x | x | x | x | x | x | x | x | x | x | x | x |  | x |  |  |  |  |  | 17 | -390.17 | 816.17 | 3.53 | 0.01 |
| 50 | x | x | x | x | x | x | x | x | x | x |  |  |  | x |  |  | x |  |  | 16 | -391.29 | 816.19 | 3.55 | 0.01 |
| 51 | x | x | x | x | x | x | x | x | x | x | x | x |  |  |  |  |  | x |  | 17 | -390.19 | 816.20 | 3.56 | 0.01 |
| 52 | x | x | x | x | x | x | x | x | x | x |  | x | x | x |  | x | x |  |  | 20 | -386.85 | 816.23 | 3.59 | 0.01 |
| 53 | x | x | x | x | x | x | x | x | x | x |  |  |  |  |  |  |  | x | x | 16 | -391.32 | 816.25 | 3.61 | 0.01 |
|  | card | aru | aru* | wb | hh | hh* | mup | mup* | wb* | edu | edu* | neigh | dist | meet | neigh* | dist* | meet* | cent | cent* | df | log(L) | AICc | ΔAICc | weight |
| 54 | x | x | x | x | x | x | x | x |  | x |  |  |  |  |  |  |  |  |  | 12 | -395.68 | 816.28 | 3.65 | 0.01 |
| 55 | x | x | x | x | x | x | x | x | x | x | x |  | x |  |  | x |  | x |  | 18 | -389.12 | 816.29 | 3.65 | 0.01 |
| 56 | x | x | x | x | x | x | x | x | x | x | x | x | x |  | x |  |  |  |  | 18 | -389.12 | 816.29 | 3.65 | 0.01 |
| 57 | x | x | x | x | x | x | x | x | x | x | x | x |  | x | x |  |  |  |  | 18 | -389.13 | 816.30 | 3.66 | 0.01 |
| 58 | x | x | x | x | x | x | x | x | x | x |  | x | x | x | x | x | x |  |  | 20 | -386.90 | 816.33 | 3.70 | 0.01 |
| 59 | x | x | x | x | x | x | x | x | x | x | x | x |  |  | x |  |  | x |  | 18 | -389.17 | 816.39 | 3.75 | 0.01 |
| 60 | x | x | x | x | x | x | x | x |  | x | x |  |  |  |  |  |  |  |  | 13 | -394.67 | 816.41 | 3.77 | 0.01 |
| 61 | x | x | x | x | x | x | x | x | x |  |  | x | x |  |  | x |  |  |  | 16 | -391.44 | 816.49 | 3.85 | 0.01 |
| 62 | x | x | x | x | x | x | x | x | x | x | x |  | x | x |  |  |  | x |  | 17 | -390.33 | 816.49 | 3.85 | 0.01 |
| 63 | x | x | x | x | x | x | x | x | x |  |  | x |  |  |  |  |  |  |  | 15 | -392.56 | 816.54 | 3.90 | 0.01 |
| 64 | x | x | x | x | x | x | x | x | x |  |  | x |  | x |  |  |  |  |  | 15 | -392.56 | 816.54 | 3.90 | 0.01 |
| 65 | x | x | x | x | x | x | x |  | x | x | x |  |  |  |  |  |  |  |  | 14 | -393.67 | 816.59 | 3.95 | 0.01 |
| 66 | x | x | x | x | x | x | x | x | x | x | x |  | x | x |  | x | x | x |  | 20 | -387.04 | 816.60 | 3.97 | 0.01 |
| 67 | x | x | x | x | x | x | x | x | x |  |  | x | x |  |  |  |  |  |  | 15 | -392.60 | 816.62 | 3.98 | 0.01 |
| RVI | 1 | 1 | 1 | 1 | .99 | .99 | .96 | .95 | .95 | .63 | .45 | .41 | .34 | .26 | .25 | .24 | .15 | .15 | .05 |  |  |  |  |  |

card: Unmatched Count Technique (UCT) card type (control or treatment)

aru: Authorized Resource User (ARU) (Yes or No)

wb: wellbeing (Average, Somewhat bad, Worst)

hh: Marital status of household head (Single or Married)

mup: Benefit from Multiple Use Program (Yes or No)

edu: Education ( 0-3 or 4+ years of formal education)

neigh: Density of neighbours (Few/none or Many)

dis: Proximity to the Park (continuous variable)

meet: Attend Park meetings (Yes or No)

cent: Nearest trading center (over or under 1 hour)

* indicates the interaction of the variable with UCT card type

**Table S4.** Set of models selected based on AICc for firewood collection from the park. x indicates that the variable was included in the model. The degrees of freedom (df), the log-likelihood (log(L)), the information criterion value (AICc), the AICc difference (ΔAICc) and AICc weight are given for each model. The Relative Variable Importance (RVI) is given at the end. Variables removed from the model during simplification were marital status (single parent household or married), ‘wellbeing’ (worst, somewhat bad or average), whether or not the respondent had ever attended a meeting with UWA, household size, and whether or not the respondent perceived themselves to have benefitted from a tree-planting, livestock provision, conservation education, health clinic or water supply project.

| Model | card | edu | center | center* | edu* | dist | dist* | crop.bft | crop.bft* | df | log(L) | AICc | ΔAICc | weight |
| --- | --- | --- | --- | --- | --- | --- | --- | --- | --- | --- | --- | --- | --- | --- |
| 1 | x | x | x | x | x |  |  |  |  | 7 | -421.03 | 856.39 | 0.00 | 0.17 |
| 2 | x | x | x | x | x | x | x |  |  | 9 | -419.53 | 857.58 | 1.18 | 0.09 |
| 3 | x | x | x | x |  |  |  |  |  | 6 | -422.83 | 857.91 | 1.51 | 0.08 |
| 4 | x | x | x | x | x |  |  | x |  | 8 | -421.01 | 858.44 | 2.05 | 0.06 |
| 5 | x | x | x | x | x | x |  |  |  | 8 | -421.01 | 858.44 | 2.05 | 0.06 |
| 6 | x | x | x | x | x |  |  | x | x | 9 | -419.96 | 858.45 | 2.06 | 0.06 |
| 7 | x | x |  |  | x | x | x |  |  | 7 | -422.18 | 858.68 | 2.29 | 0.05 |
| 8 | x |  | x | x |  |  |  |  |  | 5 | -424.28 | 858.72 | 2.33 | 0.05 |
| 9 | x | x | x | x | x | x | x | x | x | 11 | -418.02 | 858.80 | 2.41 | 0.05 |
| 10 | x | x |  |  | x |  |  |  |  | 5 | -424.50 | 859.18 | 2.78 | 0.04 |
| 11 | x | x | x | x |  | x | x |  |  | 8 | -421.54 | 859.49 | 3.10 | 0.04 |
| 12 | x | x | x | x |  |  |  | x | x | 8 | -421.60 | 859.61 | 3.22 | 0.03 |
| 13 | x | x | x | x | x | x | x | x |  | 10 | -419.52 | 859.67 | 3.28 | 0.03 |
| 14 | x | x |  |  |  |  |  |  |  | 4 | -425.79 | 859.69 | 3.30 | 0.03 |
| 15 | x | x |  |  |  | x | x |  |  | 6 | -423.77 | 859.78 | 3.38 | 0.03 |
| 16 | x | x | x | x |  |  |  | x |  | 7 | -422.81 | 859.94 | 3.55 | 0.03 |
| 17 | x | x | x | x |  | x |  |  |  | 7 | -422.81 | 859.95 | 3.55 | 0.03 |
| 18 | x | x | x | x |  | x | x | x | x | 10 | -419.85 | 860.35 | 3.96 | 0.02 |
| 19 | x |  | x | x |  | x | x |  |  | 7 | -423.02 | 860.35 | 3.96 | 0.02 |
| RVI | 1 | 0.92 | 0.84 | 0.84 | 0.63 | 0.44 | 0.35 | 0.29 | 0.17 |  |  |  |  |  |

card: Unmatched Count Technique (UCT) card type (Control or Treatment)

edu: Education ( 0-3 or 4+ years of formal education)

center: Nearest trading center (Over or under 1 hour)

dist: Proximity to the Park (Within 1 km or over 1 km)

crop.bft: Benefit from crop raiding mitigation projects (Yes or No)

* indicates the interaction between the variable and UCT card type

**Table S5**. Motivations for resource use, as ranked by 17 focus groups. Salience scores range between 0 and 1, where 1 indicates that the motivation was ranked top by focus groups in every list. A lower salience means that the motivation was ranked lower and/or not included in every list.

| Motivation for resource harvest | Salience |
| --- | --- |
| Lack of food - meat | 0.629 |
| Need medicine - herbs cure more effectively than modern medicine | 0.482 |
| Land scarcity - need firewood | 0.478 |
| Need medicine - herbs only grow in the forest | 0.403 |
| Crop raiding - anger at event and lack of compensation | 0.378 |
| Make money - honey | 0.343 |
| Make money - basketry materials | 0.322 |
| Make money - meat | 0.296 |
| Household use - basketry materials | 0.285 |
| Lack of food - honey | 0.276 |
| Lack of employment – anger and need to generate income | 0.269 |
| Crop raiding - need to replace lost income | 0.252 |
| Need medicine - honey | 0.236 |
| Crop raiding - chase animals back into the national park | 0.215 |
| Revenue sharing - anger at corruption and inequity | 0.210 |
| Make money - pit sawing | 0.202 |
| Crop raiding - no food | 0.191 |
| Household use – weaving mats | 0.164 |
| Culture - evil spirits drive people into the forest | 0.152 |
| Influence of family | 0.139 |
| Make money - handcrafts made from wood | 0.139 |
| Land scarcity - need bamboo but not enough grows outside the forest | 0.118 |
| Make money - weaving mats | 0.112 |
| Land scarcity - need timber but not enough trees growing outside the forest | 0.111 |
| Land scarcity - need building poles but not enough outside the forest | 0.110 |
| Lack of food - fish | 0.101 |
| Need medicine - meat | 0.098 |
| Make money - gold | 0.090 |
| Nowhere else - hoe handles cannot be found outside the forest | 0.083 |
| Lack of employment - idleness | 0.082 |
| Laziness | 0.078 |
| Make money for school fees | 0.078 |
| Land scarcity - need somewhere to graze animals | 0.060 |
| Crop raiding - children guard crops so lack education | 0.059 |
| Nowhere else - vines for building with cannot be found outside the forest | 0.059 |
| Culture - to access hot springs | 0.053 |
| Lack of scholarships - anger | 0.052 |
| Land scarcity - beehives in forest | 0.051 |
| Need medicine - clinic too far | 0.051 |
| Lack of food - fruit | 0.048 |
| Need medicine - faster than services at clinic | 0.041 |
| Cut tree for a beehive | 0.039 |
| Culture - our parents fed us forest food | 0.037 |
| Maintain path through Park | 0.037 |
| No access to Multiple Use Program | 0.028 |
| Make money - make a bench | 0.025 |
| Anger at death of poacher | 0.025 |
| Culture - it was our home | 0.025 |
| Nowhere else - bean stakes | 0.024 |
| Lack of food - wild yams | 0.022 |
| Lack of food - *Solanum nigrum* | 0.022 |
| Make money - fish | 0.022 |
| Growing population leads to lack of food | 0.020 |
| Culture - ancestral worship sites | 0.018 |
| Need medicine - clinic too expensive | 0.017 |
| Make money - medicine | 0.015 |
| Nowhere else - panga handles | 0.013 |
| Ignorance | 0.010 |
| Land scarcity - make charcoal | 0.007 |

**Table S6**. Deterrents against resource use, as ranked by 17 focus groups. Salience scores range between 0 and 1, where 1 means that the deterrent was ranked top by focus groups in every list. A lower salience means that the deterrent was ranked lower and/or not included in every list.

| Deterrent against resource harvest | Salience |
| --- | --- |
| Law enforcement | 0.902 |
| Sensitisation - conservation education | 0.524 |
| Influence of family | 0.456 |
| Influence of stretcher group (fine) | 0.435 |
| Benefit - tourists bring money to community and country | 0.384 |
| Benefit - schools | 0.347 |
| Influence of community | 0.331 |
| Benefit - forest brings rain | 0.288 |
| Sensitisation - now want to protect gorillas | 0.236 |
| Waste of time - have resources or money to buy them | 0.188 |
| Benefit - revenue sharing (goats) | 0.186 |
| Benefit - hope for benefits from conservation projects in the future | 0.186 |
| Benefit - climate stabilisation and oxygen | 0.166 |
| Influence of stretcher group (education) | 0.155 |
| Fear to be killed | 0.149 |
| Benefit - health clinics | 0.148 |
| Religion | 0.121 |
| Fear animals in the forest | 0.121 |
| Benefit - local employment (so the community does not go) | 0.090 |
| Benefit - Multiple Use Program | 0.084 |
| Love the forest and don't want to damage it | 0.078 |
| Benefit - revenue sharing (potatoes) | 0.059 |
| Waste of time - have resources (trees) | 0.059 |
| Benefit - local employment (employee’s family does not go) | 0.058 |
| Sensitisation - research dissemination | 0.051 |
| Forest is impenetrable | 0.047 |
| Authorized Resource User's fear unlawful arrest | 0.047 |
| Benefit - Mauritius thorn fence | 0.043 |
| Benefit - International relationships | 0.043 |
| Benefit - Children are sponsored to go to school | 0.041 |
| Sensitisation - religion | 0.039 |
| Don't want to damage relationship with Park | 0.039 |
| Don't have enough energy to go | 0.038 |
| Benefit - Buhoma village walk | 0.036 |
| Benefit - Bwindi Mgahinga Conservation Trust bought land | 0.036 |
| Rangers are foreign so are feared | 0.035 |
| Benefits - Gravity Water Flow Scheme | 0.034 |
| Fear Authorized Resource User's reporting me | 0.032 |
| Benefit - loaning scheme | 0.029 |
| Benefit - Tree planting | 0.027 |
| Benefit - hope for future employment | 0.026 |
| Benefit - local employment (employee does not go illegally) | 0.025 |
| Influence of tourists opinions | 0.023 |
| Waste of time - few animals left | 0.021 |
| Don't want children involved in illegal activity | 0.020 |
| Waste of time - have resource (bamboo) | 0.018 |
| Benefit - revenue sharing (road) | 0.016 |
| Mauritius thorn fence stops people from entering forest | 0.012 |
| Don't want to set fire to the forest when harvesting honey | 0.012 |
| Influence - stretcher group (report to authorities) | 0.009 |
| The Park was taken from us and we won’t get it back | 0.008 |

**Figure S1**. Sample Unmatched Count Technique cards, showing the control card on the left and the treatment card on the right, including the sensitive item, in this case bushmeat.


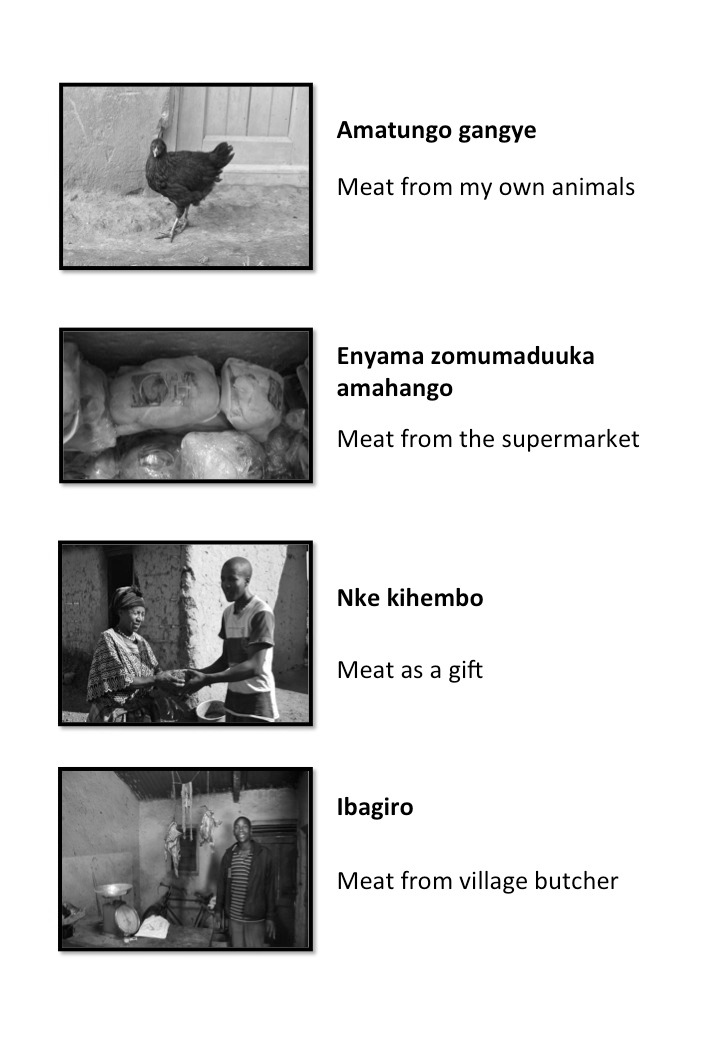

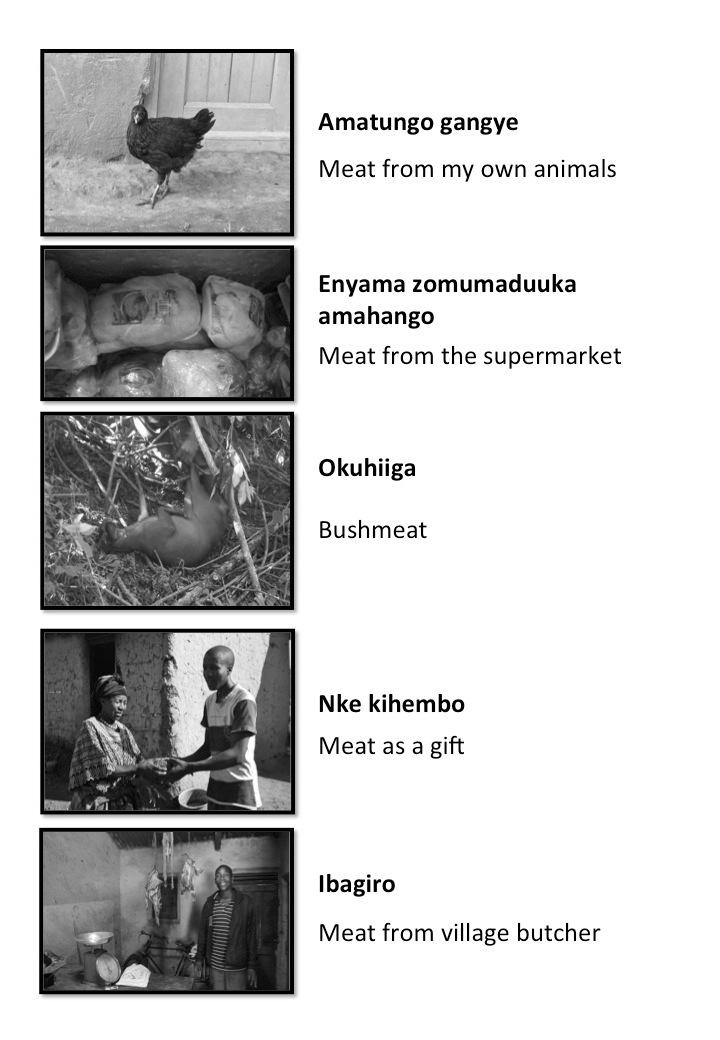

Supplement: Supplementary file 1 — Details of the survey methods, including how ethical issues were addressed, the UTC (Appendix S1), and of socioeconomic variables, UCT models, and saliences of all motivations and deterrents (Appendix S2) are available online. The authors are solely responsible for the content and functionality of these materials. Queries (other than absence of the material) should be directed to the corresponding author. Figure S1. Sample Unmatched Count Technique cards, showing the control card on the left and the treatment card on the right, including the sensitive item, in this case bushmeat. Table S1. Changes in wealth and education according to education and proximity to Bwindi Impenetrable National Park, roads and trading centers. Table S2. Variation in socioeconomic characteristics of resource user groups compared to the baseline sample mean. Table S3. Set of models selected based on AICc for bushmeat consumption. Table S4. Set of models selected based on AICc for firewood collection from the park. Table S5. Motivations for resource use, as ranked by 17 focus groups. Table S6. Deterrents against resource use, as ranked by 17 focus groups. [file COBI-29-1636-s001.docx]
